# Serum vitamin D levels, diabetes and cardio-metabolic risk factors in Aboriginal and Torres Strait Islander Australians

**DOI:** 10.1186/1758-5996-6-78

**Published:** 2014-07-16

**Authors:** Louise J Maple-Brown, Jaquelyne T Hughes, Zhong X Lu, Kanakamani Jeyaraman, Paul Lawton, Graham RD Jones, Andrew Ellis, Ashim Sinha, Alan Cass, Richard J MacIsaac, George Jerums, Kerin O’Dea

**Affiliations:** 1 Menzies School of Health Research, Charles Darwin University, Darwin, Australia; 2 Division of Medicine, Royal Darwin Hospital, Darwin, Australia; 3 Melbourne Pathology, Melbourne, Australia; 4 Department of Medicine, Monash University, Melbourne, Australia; 5 University of South Australia, Adelaide, Australia; 6 SydPath, St Vincents Hospital, Sydney, Australia; 7 University of NSW, Sydney, Australia; 8 Austin Health, Melbourne, Australia; 9 Cairns Base Hospital and Diabetes Centre, Cairns, Australia; 10 St Vincent’s Hospital, Melbourne, Australia; 11 University of Melbourne, Melbourne, Australia

**Keywords:** 25-hydroxy vitamin D, Type 2 diabetes, Aboriginal, Cardiovascular risk

## Abstract

**Background:**

Low levels of serum 25–hydroxy vitamin D (25(OH)D), have been associated with development of type 2 diabetes and cardiovascular disease (CVD); however there are limited data on serum 25(OH)D in Indigenous Australians, a population at high risk for both diabetes and CVD. We aimed to assess levels of serum 25(OH)D in Aboriginal and Torres Strait Islander Australians and to explore relationships between 25(OH)D and cardio-metabolic risk factors and diabetes.

**Methods:**

592 Aboriginal and/or Torres Strait Islander Australian participants of The eGFR (estimated glomerular filtration rate) Study, a cross-sectional analysis of a cohort study performed in 2007–2011, from urban and remote centres within communities, primary care and tertiary hospitals across Northern Territory, Far North Queensland and Western Australia. Assessment of serum 25(OH)D, cardio-metabolic risk factors (central obesity, diabetes, hypertension, history of cardiovascular disease, current smoker, low HDL-cholesterol), and diabetes (by history or HbA1c ≥6.5%) was performed. Associations were explored between 25(OH)D and outcome measures of diabetes and number of cardio-metabolic risk factors.

**Results:**

The median (IQR) serum 25(OH)D was 60 (45–77) nmol/L, 31% had 25(OH)D <50 nmol/L. For participants with 25(OH)D < 50 vs ≥50 nmol/L, cardio-metabolic risk profile differed for: diabetes (54%, 36% p < 0.001), past history of cardiovascular disease (16%, 9%, p = 0.014), waist-hip ratio (0.98, 0.92, p < 0.001), urine albumin-creatinine ratio (2.7, 1.5 mg/mmol, p < 0.001). The OR (95% CI) for diabetes was 2.02 (1.03 – 3.95) for people in the lowest vs highest tertiles of 25(OH)D (<53 vs >72 nmol/L, respectively) after adjusting for known cardio-metabolic risk factors.

**Conclusion:**

The percentage of 25(OH)D levels <50 nmol/L was high among Aboriginal and Torres Strait Islander Australians from Northern and Central Australia. Low 25(OH)D level was associated with adverse cardio-metabolic risk profile and was independently associated with diabetes. These findings require exploration in longitudinal studies.

## Background

The major contributor to the significantly reduced life expectancy of Indigenous Australians, compared to the general Australian population is the overwhelming burden of chronic diseases, such as ischaemic heart disease, diabetes and chronic kidney disease [1]. Low levels of serum 25–hydroxy vitamin D (25(OH)D), the best marker for vitamin D status, have been associated with development of the metabolic syndrome [2], diabetes [3,4] and cardiovascular disease [5] in many parts of the world, but there are limited data on serum 25(OH)D levels in the high risk Indigenous Australian population.

Most available data on vitamin D deficiency in Australia relate to studies performed in the temperate zones. The Australian Diabetes, Obesity and Lifestyle (AusDiab) study reported that 31% of adults in Australia had serum 25(OH)D levels less than 50 nmol/L, with a higher prevalence during winter–spring and in people residing in southern states [6]. Studies that have assessed vitamin D levels in groups such as the elderly [7], and free-living adults in Southeast Queensland (subtropical) [8] have demonstrated a prevalence of vitamin D deficiency (defined as serum 25(OH)D < 50 nmol/L) of at least 25%. However, small studies from the tropical region of Far North Queensland have reported a low prevalence of vitamin D deficiency in a healthy obstetric population (0%) [9] and in free-living adults (6.2%) [10]. Large population-based studies on vitamin D status are lacking in tropical Australia.

It is notable that the prevalence of vitamin D deficiency is higher in people with dark skin. A recent systematic review reported a prevalence of 30-53% in ethnic minorities with dark skin versus 14-26% in those of Europid background [11]. Limited data from South Australia show that Indigenous Australians have a low vitamin D status [12]. However, approximately 40% of Indigenous Australians live at latitudes closer to the equator - in the tropical and subtropical regions of Australia [13]. In the context of high risk of premature mortality from cardiovascular disease and related chronic diseases in Indigenous Australians and the reported association between vitamin D and chronic diseases, further assessment of the vitamin D status and its relationship to cardio-metabolic risk factors in this high risk population is warranted.

Therefore, we evaluated the serum 25(OH)D levels in Aboriginal and Torres Strait Islander Australians in a cross sectional cohort of 592 Indigenous Australians from urban, regional and remote areas of the Northern Territory, Western Australia and Far North Queensland, participants in The Estimated Glomerular Filtration Rate Study (The eGFR Study) [14,15]. The aim of the eGFR Study was to assess the accuracy of estimated measures of glomerular filtration rate in Indigenous Australians. The aim of the current analysis is to assess levels of serum 25(OH)D in Indigenous Australians and to explore relationships between 25(OH)D and cardio-metabolic risk factors in this cohort.

## Methods

### Participants

The methods of the eGFR Study have been previously reported [14,15]. In brief, Aboriginal and/or Torres Strait Islander participants aged 16 years and above were recruited across five predefined strata of health, diabetes status and kidney function from the following regions of Australia: Top End, Northern Territory, Central Australia, remote Western Australia and Far North Queensland. The study was approved by the joint Menzies School of Health Research and Northern Territory Department of Health Human Research Ethics Committee (HREC) and other HRECs in the above regions [14].

Participants fulfilled the definition of ‘Aboriginal and/or Torres Strait Islander’ according to the standard method used in National Census data collection: “1) is of Aboriginal and/or Torres Strait Islander descent; 2) identifies as an Australian Aboriginal and/or Torres Strait Islander; and 3) is accepted as such by the community in which he or she lives or has lived.” Of the 656 Indigenous participants of the eGFR Study, 605 had available data for serum 25(OH)D. Participants aged <18 years were excluded from this analysis (n = 13), leaving n = 592 participants presented here. Participants of both Aboriginal and Torres Strait Islander background (n = 52) were combined with the Torres Strait Islander group as the two groups did not differ significantly for: age, body mass index (BMI), waist circumference, waist hip ratio (WHR), blood pressure (BP), HbA1c, lipid profile, urine albumin creatinine ratio (ACR), and serum 25(OH)D. The combined group of Torres Strait Islander participants and those of both Aboriginal and Torres Strait Islander background is referred to as Torres Strait Islander participants. This analysis includes 22 participants without data for measured glomerular filtration rate (mGFR), and participants missing data for other cardio-metabolic variables.

The latitude of participants’ usual residence (Additional file 1: Figure S1) was [median, interquartile range (IQR), (range)]: Aboriginal participants, 16.4, 12.5-23.7 (11.2-35.1) degrees south, Torres Strait Islander participants 10.6, 10.6-10.6 (10.0-37.8) degrees south. Note that 66% (112 of 169) of Torres Strait Islander participants resided on Thursday Island at latitude 10.6 degrees south. The season of sampling was: Aboriginal participants, 5.4% Summer (December-February), 28.6% Autumn (March-May), 42.3% Winter (June-August), 23.6% Spring (September-November); Torres Strait Islander participants, 42.0% Summer, 4.7% Autumn, 23.1% Winter, 30.2% Spring.

### Laboratory methods

The laboratory methods have been previously published [14]. In brief, urine albumin and creatinine, lipid profile (non-fasting) and HbA1c assays were performed at each centre by the local laboratory. The mGFR was measured using an iohexol plasma disappearance technique over 4 hours [14]. Serum high sensitive troponin T (hsTnT) and 25(OH)D were measured at Melbourne Pathology, Melbourne, Australia. Serum hsTnT was analysed using the electro-chemiluminescence immunoassay on a COBAS e601 analyser (Roche Diagnostics, Mannheim) with an inter-assay coefficient variation (CV) of 3.1%. Serum 25(OH)D was analysed using the 25OH vitamin D TOTAL assay on a Liaison XL analyser (DiaSorin Inc., Stillwater, MN). It is an automatic, direct competitive chemiluminescent immunoassay with an inter-assay CV of 6.9% at 41 nmol/L and 5.8% at 101 nmol/L. This method gives serum 25(OH)D results that align with the National Institute of Standards and Technology (NIST) targets. Compared to the NIST-aligned liquid chromatography-tandem mass spectrometry system, the equation obtained is y (Liaison XL in nmol/L) = 1.03× – 1.1 [16].

### Cardio-metabolic risk factors

Cardio-metabolic risk factors included components of the metabolic syndrome, using National Cholesterol Education Program, International Diabetes Federation and harmonised definitions [17]:

(a) Central Obesity: WHR > 0.9 for males and >0.85 for females

(b) Diabetes: by history or HbA1c ≥6.5%

(c) Low HDL-Cholesterol: <1 mmol/L for males and <1.3 mmol/L for females

(d) Hypertension: systolic BP ≥ 130 mmHg or diastolic BP ≥ 85 mmHg

(e) Current smoker: current cigarette smoker

(f) Past history of cardiovascular disease (CVD): history of ischaemic heart disease, acute myocardial infarction, cerebrovascular accident or transient ischaemic attack by self-report and/or medical records.

### Statistical methods

**Figure 1:**
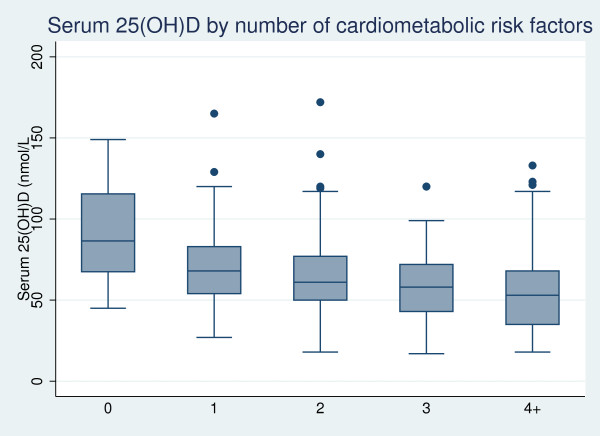
**Serum 25(OH)D distribution in participants with increasing number of cardio-metabolic risk factors (central obesity, diabetes, low HDL-cholesterol, hypertension, current smoker, past history of CVD).** The bars represent median and boxes represent interquartile range of serum 25(OH)D (nmol/L), dots represent outliers; n = 24, 87, 127, 149, 118 across groups with 0, 1, 2, 3, 4+ cardio-metabolic risk factors, respectively; p < 0.001 for each category of cardiometabolic risk numbers compared to reference group of 0 risk factors.

Data analysis was performed using STATA v12.0 (Stata Corporation, TX, USA). Data are presented stratified by 25(OH)D levels <50 and ≥50 nmol/L (Table 1) as that is the cut-point used in national and international clinical guidelines. Variables with distributions significantly different from normal were log transformed. To determine between group differences, Pearson chi-square tests (categorical variables) or independent sample t-tests (continuous variables) were conducted. Due to missing data, n of each group in Table 1 for the following variables was: BMI, 184, 405; WHR, 172, 391; BP, 184, 402; Smoker, 183, 400; History CVD, 178, 387; HbA1c, 180, 394; HDL, 177, 381; mGFR, 176, 394; urine ACR, 183, 397. Cardio-metabolic risk factors were assessed in all participants and each ethnic group. The number of cardio-metabolic risk factors was calculated by counting how many of the following six factors were present: central obesity, low HDL-cholesterol, hypertension, diabetes, current smoker, and past history of CVD. Due to missing data, n of all participants and each group respectively in Table 2, for the following variables was: central obesity, 563, 398, 165; low HDL, 558, 402, 156; hypertension, 586, 419, 167; past history CVD, 565, 400, 165; current Smoker, 583, 417, 166; sum of CVD Risk Factors, 505, 358, 147. Distribution of 25(OH)D in participants with increasing number of cardio-metabolic risk factors was assessed graphically using a box plot (Figure 1). Logistic regression was used to estimate the odds ratio for participants in tertiles of serum 25(OH)D for two outcome variables: three or more cardio-metabolic risk factors (3+ cardio-metabolic risk factors) and diabetes. Models were performed using tertiles of 25(OH)D. Three models were performed for each outcome variable. Models 1 and 2 were the same for both outcome variables: model 1 was unadjusted; and model 2 adjusted for age, gender, ethnicity (Aboriginal or Torres Strait Islander), season and latitude. Different covariables were used in model 3. For the relationship between vitamin D and 3 + cardio-metabolic risk factors, model 3 was model 2 further adjusted for mGFR, troponin and urine ACR. For the relationship between vitamin D and diabetes, model 3 was model 2 further adjusted for WHR, HDL-cholesterol, mGFR, systolic blood pressure, current smoker, urine ACR. The fully adjusted models were constructed including covariates if they were clinically important or confounders. Tertiles of serum 25(OH)D were used in the multivariate modelling in order to perform a robust analysis of relationships between key variables, without an a priori decision as to where cut-points should be. For each outcome variable, two-way interactions were assessed between vitamin D and other independent variables in the model. There were no significant interaction terms.

**Table 1:** Baseline characteristics stratified by serum 25(OH)D

	**25(OH)D <50 nmol/L**	**25(OH)D ≥50 nmol/L**	p value*
*n*	*186*	*406*	*-*
Age (years)	46 (12)	45 (15)	0.35
Aboriginal (%)	168 (90%)	255 (63%)	
Torres Strait Islander (%)	18 (10%)	151 (37%)	**<0.001**
Male (%)	71 (38%)	150 (37%)	0.78
Current smoker (%)	77 (42%)	169 (42%)	0.97
Diabetes (%)	100 (54%)	148 (36%)	**<0.001**
Past History CVD (%)	28 (16%)	34 (9%)	**0.014**
BMI (kg/m^2^)	30.5 (7.1)	29.5 (7.3)	0.15
Waist Hip ratio	0.97 (0.09)	0.92 (0.09)	**<0.001**
Systolic BP (mmHg)	122 (19)	115 (16)	**<0.001**
Diastolic BP (mmHg)	77 (11)	73 (10)	**<0.001**
25(OH)D (nmol/L)	37 (9)	74 (20)	**<0.001**
HbA1c (%, mmol/mol)	6.3 (5.9-7.8) 45 (40–62)	5.9 (5.5-6.7) 41 (37–50)	**<0.001**
HDL chol (mmol/L)	1.0 (0.8-1.2)	1.1 (0.9-1.3)	0.29
Total chol: HDL chol	4.5 (3.6-5.8)	4.4 (3.6-5.7)	0.21
Troponin (μg/L)	3.0 (3.0-5.2)	3.0 (3.0-5.7)	0.10
mGFR (ml/min/1.73 m^2^)	104 (76–123)	102 (84–119)	0.50
mGFR < 60 ml/min/1.73 m^2^ (%)	39 (21%)	63 (16%)	0.10
Urine ACR (mg/mmol)	2.7 (0.9-81)	1.5 (0.7-10)	**<0.001**

Data presented as mean (standard deviation), median (interquartile range) or number (%).

n may vary due to missing data (see methods).

*Pearson chi-square test or independent sample t-test.

p values <0.05 are represented in bold font.

**Table 2:** Number (%) of Aboriginal and Torres Strait Islander participants with cardio-metabolic risk factors

**Risk factor**	**All participants**		**Aboriginal**		**Torres Strait Islander**	
	***n = 592***		***n = 423***		***n = 169***	
	*Male (n==221)*	*Female (n = 371)*	*Male (n==152)*	*Female (n = 271)*	*Male (n = 69)*	*Female (n = 100)*
Central Obesity	163 (76%)	262 (75%)	116 (79%)	200 (80%)	47 (69%)	62 (64%)
Diabetes	86 (39%)	162 (44%)	64 (42%)	121 (45%)	22 (32%)	41 (41%)
Low HDL*	100 (47%)	249 (72%)	63 (43%)	185 (72%)	37 (56%)**	64 (71%)
Hypertension*	69 (32%)	83 (23%)	47 (31%)	69 (26%)	22 (33%)	14 (14%)
Past History CVD	30 (14%)	32 (9%)	24 (17%)	30 (12%)	6 (9%)**	2 (2%)
Current smoker	100 (46%)	146 (40%)	72 (48%)	111 (42%)	28 (42%)	35 (35%)
**3+ Cardio-metabolic Risk Factors**	97 (51%)	170 (54%)	69 (53%)	130 (57%)	28 (46%)	40 (47%)
**4+ Cardio-metabolic Risk Factors**	46 (24%)	72 (23%)	32 (24%)	58 (26%)	14 (23%)	14 (16%)

Data are n (%).

Where 3+ and 4+ CVD risk factors includes the 6 risk factors outlined in the above table.

n may vary due to missing data (see methods).

*Among all participants, proportions differed by gender for low-HDL (p < 0.001) and hypertension (p = 0.015).

**p < 0.05 between Aboriginal and Torres Strait Islander participants (Pearson chi-square test).

## Results

Of the 592 Indigenous participants, 423 (71%) identified as Aboriginal and 169 (29%) as Torres Strait Islander. The median (IQR) serum 25(OH)D was: all Indigenous participants, 60 (45–77) nmol/L; Aboriginal participants, 55 (40–70) nmol/L; Torres Strait Islander participants, 75 (58–88) nmol/L. Among all participants (n = 592), the distribution of serum 25(OH)D <50, 50–74 and ≥75 nmol/L was 31%, 42% and 27% respectively. Participant characteristics, stratified by serum 25(OH)D <50 or ≥50 nmol/L, are presented in Table 1. A higher proportion of Aboriginal participants had serum 25(OH)D <50 nmol/L than Torres Strait Islander participants. Compared to participants with serum 25(OH)D ≥50 nmol, those with 25(OH)D <50 nmol/L displayed a more adverse cardio-metabolic risk profile: higher percentage with diabetes and past history of CVD; higher waist-hip ratio, blood pressure, HbA1c and urine ACR. The difference in urine ACR between groups with 25(OH)D < or ≥50 nmol remained significant after adjustment for diabetes status (p = 0.002) or HbA1c (p = 0.004).

Table 2 shows that 53% of all participants had at least three and 23% had at least four cardio-metabolic risk factors. Only 24 participants had nil risk factors. Levels of serum 25(OH)D fell with increasing numbers of cardio-metabolic risk factors (Figure 1). The study population was grouped on the basis of tertiles of 25(OH)D values (≥72, 53–71 and < 53 nmol/L). Compared to participants with serum 25(OH)D at the highest tertile (≥72 nmol/L), the odds ratio and 95% confidence interval (OR (95% CI)) of having 3 + cardio-metabolic risk factors was 1.95 (1.20–3.16) and 4.00 (2.35–6.82) in participants with serum 25(OH)D in the middle and lowest tertiles, respectively, after adjustment for age, gender, ethnicity (Aboriginal or Torres Strait Islander), latitude and season. The strength of the association was reduced but still remained significant after inclusion of mGFR, troponin and urine ACR as covariates in the above model [OR (95% CI) 1.69 (1.02–2.81) for 25(OH)D 53–72 nmol/L and 3.04 (1.71–5.40) for 25(OH)D <53 nmol/L].

Table 3 outlines the OR (95% CI) for the association between each tertile of 25(OH)D and diabetes in different models. Compared to participants in the highest tertile of serum 25(OH)D (>72 nmol/L), the risk of diabetes for participants in the lowest tertile (<53 nmol/L) was doubled [OR (95% CI) 2.15 (1.10 – 2.18)] after adjusting for cardio-metabolic risk factors in model 3.

**Table 3:** Risk of diabetes expressed as odds ratio according to tertiles of serum 25(OH)D levels

**25(OH)D nmol/L**	**Model 1**	**Model 2**	**Model 3**
	***n = 592***	***n = 592***	***n = 489***
T1 (≥72)	Ref	Ref	Ref
T2 (53–71)	1.80 (1.18 - 2.75)	1.73 (1.06 – 2.83)	1.56 (0.84 – 2.90)
T3 (<53)	2.80 (1.85 – 4.23)	3.01 (1.81 – 5.03)	2.15 (1.10 – 2.18)
**Covariates in the model**	Unadjusted	Age, gender, ethnicity (Aboriginal or Torres Strait Islander), season, latitude	All co-variates in Model 2 and waist-hip ratio, HDL-cholesterol, mGFR, systolic BP, current smoker, urine ACR

Results presented are those of logistic regression analysis of diabetes.

## Discussion

In this large cross-sectional study of 25(OH)D levels in Aboriginal and Torres Strait Islander Australians from Northern and Central Australia, we have reported three key findings. First, one third (31%) of this Indigenous cohort had vitamin D deficiency (serum 25(OH)D <50 nmol/L). Second, Indigenous Australian participants with 25(OH)D levels less than 50 nmol/L displayed a more adverse cardio-metabolic risk profile than those with 25(OH)D level greater than 50 nmol/L. Third, serum 25(OH)D levels in the lowest tertile (<53 nmol/L) were independently associated with diabetes, and the association remained significant after adjustment for known cardio-metabolic variables.

To our knowledge, this is the first large study of 25(OH)D levels in adult Aboriginal and Torres Strait Islander Australians. We have reported a high percentage (31%) of vitamin D deficiency in this cohort from Northern and Central Australia, regions closer to the equator than previous Australian studies. The median 25(OH)D level (55 nmol/L) is similar to the mean 25(OH)D previously reported in a small study of 58 Aboriginal participants from South Australia (latitude 35 degrees south), of 56.8 nmol/L [12]. Of note, AusDiab, a nation-wide Australian population-based study, reported that 31% of Australians had 25(OH)D levels <50 nmol/L, however only 0.9% of AusDiab participants identified as Aboriginal or Torres Strait Islander Australians [6]. High rates of vitamin D deficiency have been reported in other Indigenous populations internationally, including Aboriginal Canadian women (33%) [18], New Zealand Maori and Pacific Islander New Zealanders (61% and 69% respectively, compared to 46% of New Zealanders of European descent) [19]. However, direct comparison is limited as key characteristics (latitude, age, BMI, co-morbidities) differ between cohorts.

Aboriginal and Torres Strait Islander participants in the current study with levels of 25(OH)D less than 50 nmol/L displayed a more adverse cardio-metabolic risk profile than those with 25(OH)D greater than 50 nmol/L. Levels of 25(OH)D in the lowest tertile (<53 nmol/L) remained significantly associated with at least three cardio-metabolic risks independent of age, gender, ethnicity (Aboriginal or Torres Strait Islander), latitude, season, urine ACR, mGFR and troponin. Waist-hip ratio was used as the index of central obesity (rather than waist circumference) as it is a better discriminator of CVD risk across populations of different body build [20]. The relationship between 25(OH)D levels and cardio-metabolic risk factors observed in the current study is consistent with that described in large Australian and international observational studies, both cross-sectional [21] and longitudinal, for the outcome of cardiovascular events [5] and metabolic syndrome [2].

Low serum 25(OH)D was significantly associated with increased diabetes risk in this cross-sectional cohort. This is independent of other known cardio-metabolic variables (including waist-hip ratio, HDL-cholesterol, urine ACR, mGFR, blood pressure and cigarette smoking). A similar independent relationship between diabetes and 25(OH)D levels has been reported in other cross-sectional studies [11], and confirmed in prospective observational studies [3,4]. However, intervention studies have not clearly demonstrated efficacy of vitamin D supplementation against development of type 2 diabetes [22]. Several mechanisms have been proposed for the role of vitamin D in diabetes. Adequate serum 25(OH)D levels promotes synthesis of 1,25(OH)_2_D in extra-renal tissues. Improved beta-cell function by 1,25(OH)_2_D produced by pancreatic beta-cells, improved insulin sensitivity by 1,25(OH)_2_D in skeletal muscle, liver and adipose tissue, and systemic anti-inflammatory effects of 1,25(OH)_2_D reduce the risk of developing diabetes [4,23]. The odds ratio of the association between low vitamin D and diabetes decreased with addition of covariates (including season, latitude, and diabetes and cardio-metabolic risk factors) to the model. Data for physical activity and sun exposure were not available in the current study; inclusion of these data may have further attenuated the reported association, although data for central obesity and latitude were included.

High levels of vitamin D deficiency in Aboriginal and Torres Strait Islander participants in the current study may be related to darker skin pigmentation, reduced hours of sun exposure and/or change in physical activity levels from the hunter-gatherer to the sedentary indoor lifestyle. The change in dietary pattern from traditional (which was nutrient rich and included vitamin D-rich offal and a variety of marine species) to a Western pattern could also be a contributory factor, however dietary sources of vitamin D are often relatively low. The majority of Australians do not meet the recommended dietary intake of vitamin D [24] and vitamin D fortification is voluntary for dairy products (and mandatory only for edible oil spreads) in Australia.

Limitations of the current study include: its cross-sectional design; data were not collected for physical activity, vitamin D supplementation, dietary sources of vitamin D, skin pigmentation, sun exposure, time spent outdoors, sun protection behaviour; differences between Aboriginal and Torres Strait Islander participants for proportion with diabetes or CVD, latitude and season of blood collection. Season was categorised as the traditional four Australian seasons, as was used in the AusDiab study, although available data indicate that hours of sunlight per season vary less in tropical than in subtropical regions of this study [25]. Nevertheless, this is the largest study reporting 25(OH)D levels in Aboriginal and Torres Strait Islander Australians, a population at high risk of diabetes and CVD. Participants were from tropical and subtropical regions of Australia, regions where a significant proportion of Indigenous Australians reside, yet levels of 25(OH)D have not previously been reported among Indigenous Australians from these regions.

## Conclusions

We have reported a high percentage of vitamin D deficiency among Aboriginal and Torres Strait Islander Australians from Northern and Central regions of Australia. Low level of 25(OH)D was associated with more adverse cardio-metabolic risk profile and was independently associated with diabetes in this cross-sectional cohort. Further exploration of these findings is required in both longitudinal, observational and interventional studies.

## Competing interests

All the authors declared no competing interests.

## Authors’ contributions

LMB, JH, PL, GRDJ, AE, AS, AC, RJM, GJ, and KOD designed and conducted the eGFR study. LMB designed and performed the statistical analyses guided by ZL, GJ and KOD. ZL supervised laboratory measurement of 25(OH)D. LMB drafted the manuscript with assistance from KJ, and all authors contributed important intellectual content. All authors approved the final version for publication.

## Supplementary Material

Additional file 1: Figure S1 Latitude of participants’ usual place of residence. The Numbers inside the markers indicate the number of participants from each location, the letters indicate the location (from most northerly latitude) as follows: A, Thursday Island & Torres Strait Islands; B, Elcho Island & North Arnhemland; C, Darwin region; D, Kalumbaru; E, Katherine region; F, One Arm Point & Broome region; G, Cairns & Townsville; H, Halls Creek region; I, Lajamanu; J, Tennant Creek & Barkly region; K, Port Headland region; L, Nyrippi; M, Alice Springs region; N, Brisbane; O, Geraldton region; P, Kalgoorlie region; Q, Perth region. Click here for file
