# Supplementary figures and images for: Serum vitamin D levels, diabetes and cardio-metabolic risk factors in Aboriginal and Torres Strait Islander Australians
Source: Diabetol Metab Syndr. 2014 Jul 16;6:78. doi: 10.1186/1758-5996-6-78 (PMC4155864; doi:10.1186/1758-5996-6-78)

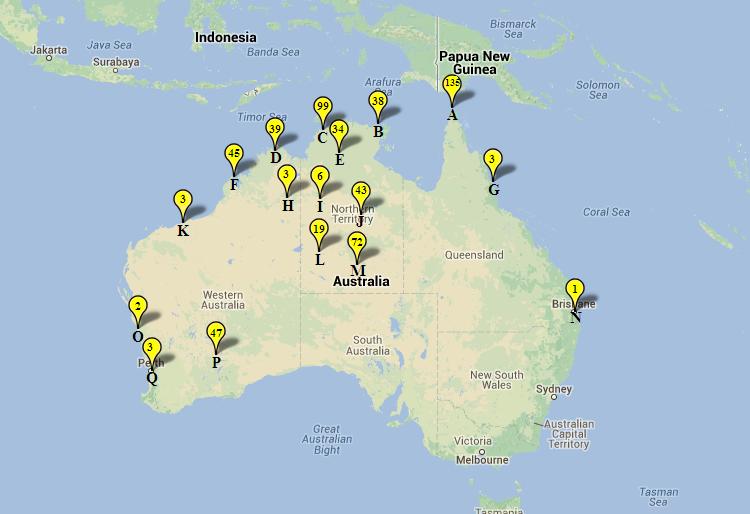

Supplement: Additional file 1: Figure S1 — Latitude of participants’ usual place of residence. The Numbers inside the markers indicate the number of participants from each location, the letters indicate the location (from most northerly latitude) as follows: A, Thursday Island & Torres Strait Islands; B, Elcho Island & North Arnhemland; C, Darwin region; D, Kalumbaru; E, Katherine region; F, One Arm Point & Broome region; G, Cairns & Townsville; H, Halls Creek region; I, Lajamanu; J, Tennant Creek & Barkly region; K, Port Headland region; L, Nyrippi; M, Alice Springs region; N, Brisbane; O, Geraldton region; P, Kalgoorlie region; Q, Perth region. [file 1758-5996-6-78-S1.jpeg]
